# Integrated analysis of gut metabolome, microbiome, and brain function reveal the role of gut-brain axis in longevity

**DOI:** 10.1080/19490976.2024.2331434

**Published:** 2024-03-28

**Authors:** Bin Jiao, Ziyu Ouyang, Qianqian Liu, Tianyan Xu, Meidan Wan, Guangrong Ma, Lu Zhou, Jifeng Guo, Junling Wang, Beisha Tang, Zhixiang Zhao, Lu Shen

**Affiliations:** a Department of Neurology, Xiangya Hospital, Central South University, Changsha, China; b National Clinical Research Centre for Geriatric Disorders, Xiangya Hospital, Central South University, Changsha, China; c Engineering Research Centre of Hunan Province in Cognitive Impairment Disorders, Central South University, Changsha, China; d Hunan International Scientific and Technological Cooperation Base of Neurodegenerative and Neurogenetic Diseases, Xiangya Hospital, Central South University, Changsha, China; e Hunan Key Laboratory of Aging Biology, Xiangya Hospital, Central South University, Changsha, China; f Department of Dermatology, Xiangya Hospital, Central South University, Changsha, China

**Keywords:** Gut metabolome, gut microbiome, brain function, gut-brain axis, longevity

## Abstract

The role of microbiota-gut-brain axis in modulating longevity remains undetermined. Here, we performed a multiomics analysis of gut metagenomics, gut metabolomics, and brain functional near-infrared spectroscopy (fNIRS) in a cohort of 164 participants, including 83 nonagenarians (NAs) and 81 non-nonagenarians (NNAs) matched with their spouses and offspring. We found that 438 metabolites were significantly different between the two groups; among them, neuroactive compounds and anti-inflammatory substances were enriched in NAs. In addition, increased levels of neuroactive metabolites in NAs were significantly associated with NA-enriched species that had three corresponding biosynthetic potentials: *Enterocloster asparagiformis*, *Hungatella hathewayi* and *Oxalobacter formigenes*. Further analysis showed that the altered gut microbes and metabolites were linked to the enhanced brain connectivity in NAs, including the left dorsolateral prefrontal cortex (DLPFC)-left premotor cortex (PMC), left DLPFC-right primary motor area (M1), and right inferior frontal gyrus (IFG)-right M1. Finally, we found that neuroactive metabolites, altered microbe and enhanced brain connectivity contributed to the cognitive preservation in NAs. Our findings provide a comprehensive understanding of the microbiota-gut-brain axis in a long-lived population and insights into the establishment of a microbiome and metabolite homeostasis that can benefit human longevity and cognition by enhancing functional brain connectivity.

## Introduction

Human longevity is a complex phenotype influenced by various factors such as genetics and environment.^1,2^ Recently, the gut microbiota has been recognized as an important factor in longevity.^3^ The composition of the gut microbiota differs across a wide range of age groups,^4^ and the distinct gut microbiota such as *Akkermansia*, *Alisipes*, and *Parabacteroides* have been reported to be associated with longevity.^4–7^ Besides, a recent study showed that the gut microbiota of centenarians exhibited youth-similar features,^7^ and mice transplanted with microbiota from long-living people exhibited a reduction in aging markers,^8^ indicating that the altered gut microbiota plays a key role in longevity.

However, the mechanisms underlying the role of the gut microbiome in healthy aging remain unclear. Gut microbiota can affect their hosts through multiple mechanisms,^9^ one of which involves metabolites.^10^ Metabolomics provides a functional readout of microbial activity and can serve as an intermediate phenotype that mediates the interactions between the gut microbiome and human health.^11^ Specific metabolites have been reported in long-lived individuals.^1,12,13^ Sato et al. found enrichment of lithocholic acid derivatives in the feces of centenarians, which were able to inhibit the growth of pathogenic bacteria and could be synthesized by bacteria isolated from centenarians, such as *Bacteroidales*.^14^ Another study showed that long-lived people successfully maintained high levels of anti-inflammatory substances in the serum, such as pinane thromboxane A2 (PTX2), which is associated with an abundance of *Alistipes* .^15^ In addition, the increased uniqueness of the gut flora, which reflects healthy aging and predicts survival in older adults, has been associated with the enrichment of beneficial metabolites such as indole in centenarians.^16^ Considering that the gut microbiota could be influenced by the limited sample size and varied ethnic regions, in this study, we first aimed to explore whether there were other altered microbiota and metabolites in a long-lived population from Central South China.

Although both microbes and metabolites are altered in long-lived people, whether this alteration is associated with functional connectivity remains unclear.^17^ Previous studies found that alterations in the gut flora have a wide range of effects on the central nervous system (CNS).^18^ Previous studies on the communication between gut bacteria and human brain, which is called the microbiota-gut-brain axis, have been focused on the pathogenesis of diverse neurological diseases.^19–21^ Brain function in long-lived people is also of great scientific interest, as some preserve cognitive function or delay the onset of dementia until advanced age.^22^ However, few studies have evaluated brain functional connectivity in long-lived individuals, and whether it is associated with the gut microbiota and metabolites is also unclear. Functional near-infrared spectroscopy (fNIRS) is a simple, fast, and portable method for brain function detection which has been applied in the research of many nervous system disease,^23–25^ and was firstly employed in the long-lived population in this study. Thus, we believe our study is the first to link gut microbes and metabolites with brain function in a long-lived population. We anticipate that an integrated multiomics approach incorporating measures of brain function will contribute to a better understanding of the specific effects of microbes on the nervous system associated with longevity.

This study integrates multidimensional datasets from 83 nonagenarians (NAs) and 81 non-nonagenarians (NNAs). Shotgun metagenomic sequencing, untargeted mass spectrometry-based metabolomics, and resting-state fNIRS (rs-fNIRS) were also performed. Informative analysis of the comprehensive phenotypic characterization enables a detailed assessment of the brain-gut connection and can provide a comprehensive overview of the microbiota-gut-brain axis in longevity.

## Results

### Demographic information of NAs and NNAs

The age ranges of NAs and NNAs were 90–102 and 55–84 years, with a median age of 94 ± 2 years and 65 ± 7 years, respectively, which were significantly different between the two groups (*p* < .001). The sex ratio (43% versus 56%) and averaged body mass index (22.58 ± 1.28 kg/m versus 23.07 ± 2.10 kg/m) were similar between NAs and NNAs(*p* > .05). There was a significant difference in education years (2.5 ± 3.3 versus 7.1 ± 2.9 years) MMSE (15.6 ± 7.1 versus 23.6 ± 5.8) and CDR (0.8 ± 0.9 versus 0.3 ± 0.6) between NAs and NNAs (*p* < .001) (Table S1).

### Fecal metabolomic profiling reveals an apparent disparity between NAs and NNAs

**Figure 1.**
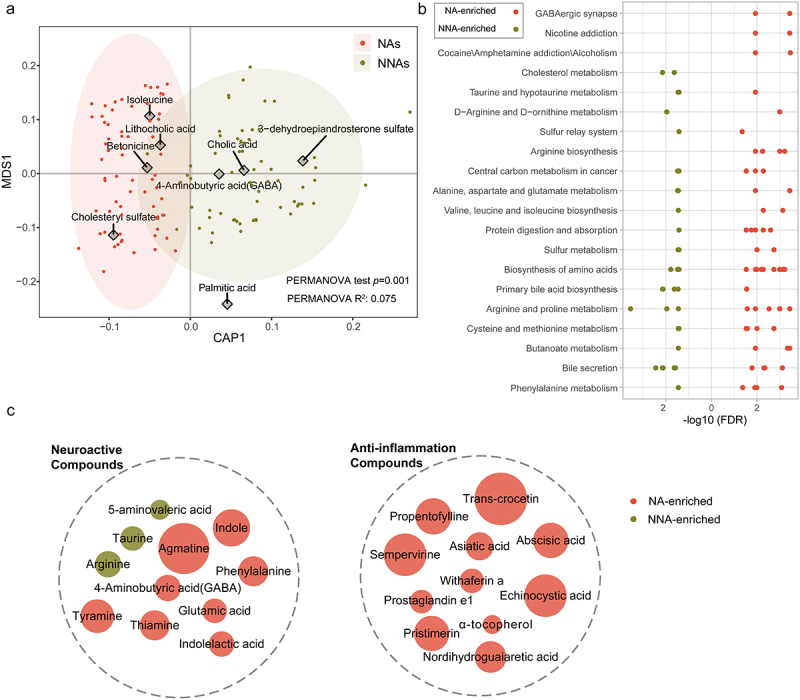
Distinct fecal metabolites associated with nonagenarians.

A total of 131 fecal samples were used for metabolomic analysis (NA, *n* = 67, NNA, *n* = 64). After untargeted LC-MS analysis, 1864 metabolites were identified, 438 of which were significantly altered in the NAs (*p* < .05), and 277 metabolites could be annotated using the KEGG database. We further applied distance-based redundancy analysis (dbRDA) to estimate the relative contribution of age characteristics to metabolite variation, and found an apparent difference between the metabolites of NAs and NNAs (Figure 1a) (PERMANOVA, *p* < .01). The variable importance in the projection (VIP) values were then calculated to evaluate the importance of each metabolite. Totally, 129 metabolites with VIP values greater than 1 were screened and defined as significantly different between the two groups, including 94 upregulated and 35 downregulated metabolites (Figure S1). Pathway enrichment analysis revealed that NA-related metabolites were involved in 100 metabolic pathways, including GABA synapses, arginine and proline metabolism, and taurine and hypotaurine metabolism et al.. (Figure 1b, Table S2). Among NA-related metabolites, the upregulated neuroactive compounds and anti-inflammatory substances included GABA, glutamic acid, agmatine and α-tocopherol, asiatic acid, and trans-crocetin (Figure 1c).

(a) Separation of fecal metabolome between NAs and NNAs, revealed by dbRDA. The metabolites identified as the major contributors to the separation are indicated by diamonds. (b) Important metabolic pathways involved in the NA-related metabolites (c) Two types of significant alterations among NA-related metabolites.

### Taxonomic and functional characterization of the gut microbiota in NAs

**Figure 2.**
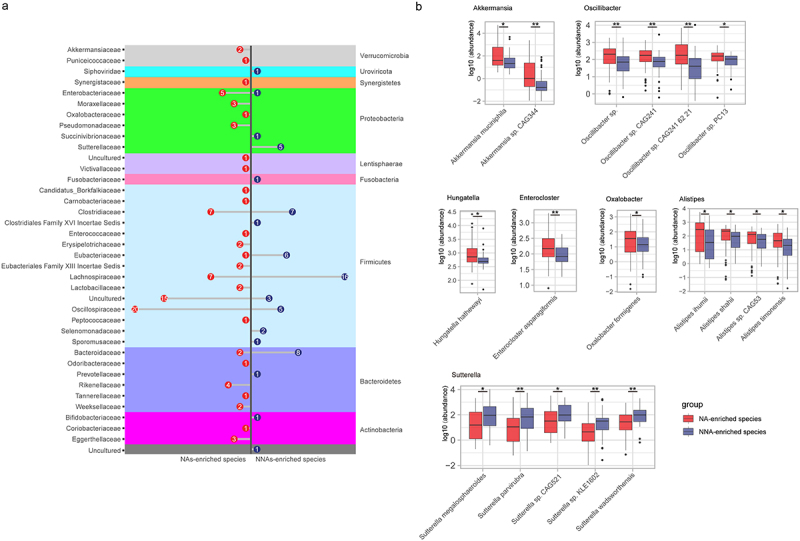
Gut microbiota species associated with nonagenarians.

In total, 87 fecal samples (NA, *n* = 50, NNA, *n* = 37) were sequenced by metagenomic shotgun sequencing. An average of 48.1 million reads (6.8Gb of data) per sample was obtained by sequencing on the Illumina platform. All reads were assembled into 3,000,000 non-redundant unigenes, which were annotated to 16,528 species and matched to 3,228 genes encoding proteins or with putative functions. The NAs had a significantly higher species count (*p* < .01) and Shannon index (*p* < .05) than NNAs (Figure S2a, b), suggesting relatively increased diversity and richness of the gut microbiota in Nas group. We also observed a higher β-diversity between groups (Figure S2c) (*R* = .06, *p* < .05), indicating that the within-group variation was higher than between-group differences. The dbRDA showed that the taxonomic composition and functional potential of the NAs microbiota differed markedly from those of the NNAs (Figure S2d, e). According to the LEfSe analysis, 153 species were significantly different between the two groups (Figure 2a, Table S3).

(a) Statistically significant results of the LEfSe analyses. Per microbial family, the number of increased (red) or decreased (green) species are shown respectively. (b) The boxplots show the prominent species that differ significantly in abundance between NAs and NNAs. Differences in abundances between the two groups were tested by two-tailed Wilcoxon’s rank-sum test and “*” stands for *p*< .05, “**” stands for *p*< .01.

Microbes that increased in NAs group mainly included *Akkermansia* (*Akkermansia muciniphila* and *Akkermansia sp. CAG344*), *Hungatella* (*Hungatella hathewayi*), *Enterocloster* (*Enterocloster asparagiformis*), *Oscillibacter* (*Oscillibacter sp*., *Oscillibacter sp. CAG241*, *Oscillibacter sp. CAG241-62-21* and *Oscillibacter sp. PC13*), *Oxalobacter* (*Oxalobacter formigenes*), and *Alistipes* (*Alistipes ihumii*, *Alistipes shahii*, *Alistipes sp. CAG53*, and *Alistipes timonensis*). While, microbes that decreased in NAs group mainly included *Sutterella* (*Sutterella megalosphaeroides*, *Sutterella parvirubra*, *Sutterella sp. CAG521*, *Sutterella sp. KLE1602* and *Sutterella wadsworthensis*) (Figure 2b). Functional analysis of the metagenomes based on KEGG pathways showed that functions related to the nervous system, including butanoate metabolism, Vitamin B6 metabolism and riboflavin metabolism were significantly different between NAs and NNAs (Table S4).

We grouped previously mentioned differential metabolites into six clusters, three was enriched in NAs and three were enriched in NNAs. Spearman analysis showed that most microbe-related functions like carbohydrate metabolism, lipid metabolism, amino acid metabolism were significantly associated with differential metabolite clusters (Figure S3), suggesting that that gut microbials may mediate metabolites changes.

### Microbiota alterations correlate with fecal metabolome changes in NAs

**Figure 3.**
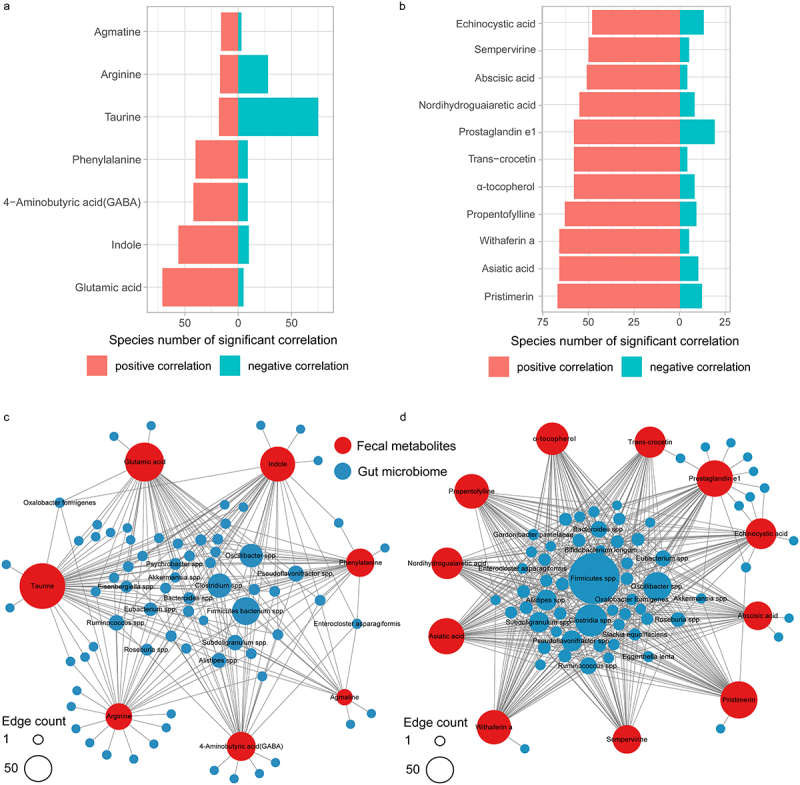
Correlation between gut microbes and neuroactive/anti-inflammatory metabolites.

To investigate the relationship between gut microbes and fecal metabolites, we performed Procrustes analysis, which showed good agreement between microbes and metabolites (Figure S4) (M^2^  = 0.44, *p* < .01). Spearman’s analysis showed a wide range of connections between differential microbes and metabolites (Figure 3a,b), especially

(a) Number of gut microbes significantly associated with neuroactive metabolites. (b) Number of gut microbes significantly associated with anti-inflammatory metabolites. (c) NA-related gut microbes and neuroactive metabolite networks. (d) NA-related gut microbes and anti-inflammatory metabolite networks.

several representative metabolites that have the potential neuroactive and anti-inflammatory effects, such as neurotransmitters (GABA and glutamic acid), indole, taurine, agmatine, arginine, phenylalanine, abscisic acid and α-tocopherol. Microbes like *Firmicutes bacterium*, *Clostridium*, and *Oscillibacter* were strongly correlated with neuroactive and anti-inflammatory substances (Figure 3c, d).

To further explore the potential mechanism by which microbes mediate changes in metabolites, we focused on the key enzyme-coding genes/pathways involved in neuroactive substance synthesis. A total of 674 species contained key enzyme-coding genes and pathways (Table S5). The distributions of the overall abundance of key enzyme-coding genes/pathways in NAs and NNAs were consistent with those of the corresponding metabolites except for phenylalanine; NA-enriched species such as *Enterocloster asparagiformis* contributed to the increase of genes/module abundances for synthesizing glutamic acid in NAs (Figure S5).

Additionally, the abundance of key enzyme-coding genes and pathways in specific species was significantly associated with the corresponding abundance of neuroactive substances. Some NA-enriched species enhanced the ability to synthesize NA-increased neuroactive substances, such as the association between *Oxalobacter formigenes* and GABA, *Enterocloster asparagiformis* and glutamic acid, *Oxalobacter formigenes*/*Hungatella hathewayi* and agmatine (Figure S6), indicating that NA-related species can affect the abundance of neuroactive substances by regulating their synthesis.

### Resting-state functional connectivity patterns in NAs

**Figure 4.**
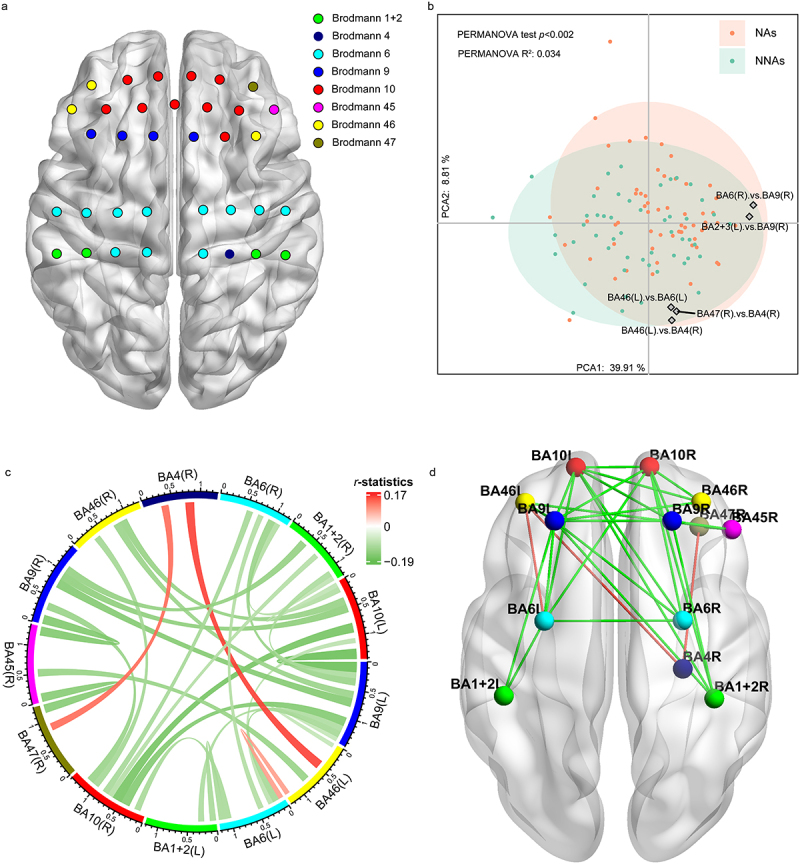
Brain functional connectivity associated with nonagenarians.

After excluding participants who were unable to finish detection due to bedridden and those with unqualified fNIRS data, finally, the fNIRS data of 60 NAs and 50 NNAs were analyzed to investigate the relationship between microbes/metabolites and brain function. A total of 1225 (35 × 35) channel-based functional connectivity pairs and an RRC matrix containing 169 (13 × 13) pairs of regions of interest (ROI)-based functional connectivity were generated for each participant (Table S6). Principal component analysis (PCA) showed that the RRC matrix of NAs was significantly different from that of NNAs (Figure 4a) (R^2^ = 0.03, *p* < .01). Differential analysis showed that 157 pairs of channel-based functional connectivity and 29 pairs of ROI-based functional connectivity were significantly different between the two groups (Table S7, S8) (FDR <0.05). Most functional connectivity was weakened in NAs, while connectivity between the left part of Brodmann’s area 46 (BA46L) and BA6L/BA4R as well as between BA47R and BA4R was strengthened in NAs (Figure 4b).

(a) Channels and ROIs map. (b) Separation of brain functional connectivity between NAs and NNAs, revealed by PCA. Arrows indicate the functional connections identified as the major contributors. (c) Chord diagram of NAs-NNAs contrasts. The shade of connection curves corresponds to respective *r*-statistics. (d) Brain map of NAs-NNAs contrasts. Significant connections (FDR <0.05) are shown as colored curves (red :NAs > NNAs; green : NAs < NNAs).

### NA-related fecal metabolites and gut microbes are tightly linked to brain functional connectivity

We evaluated the effects of microbes and metabolites on the brain functional connectivity network. Procrustes analysis showed that both microbes and metabolites were weakly but significantly correlated with brain connectivity (Figure S7)(M^2^  = 0.90, *p* < .01; M^2^  = 0.89, *p* < .01, respectively), suggesting that both could affect brain functional connectivity.

**Figure 5.**
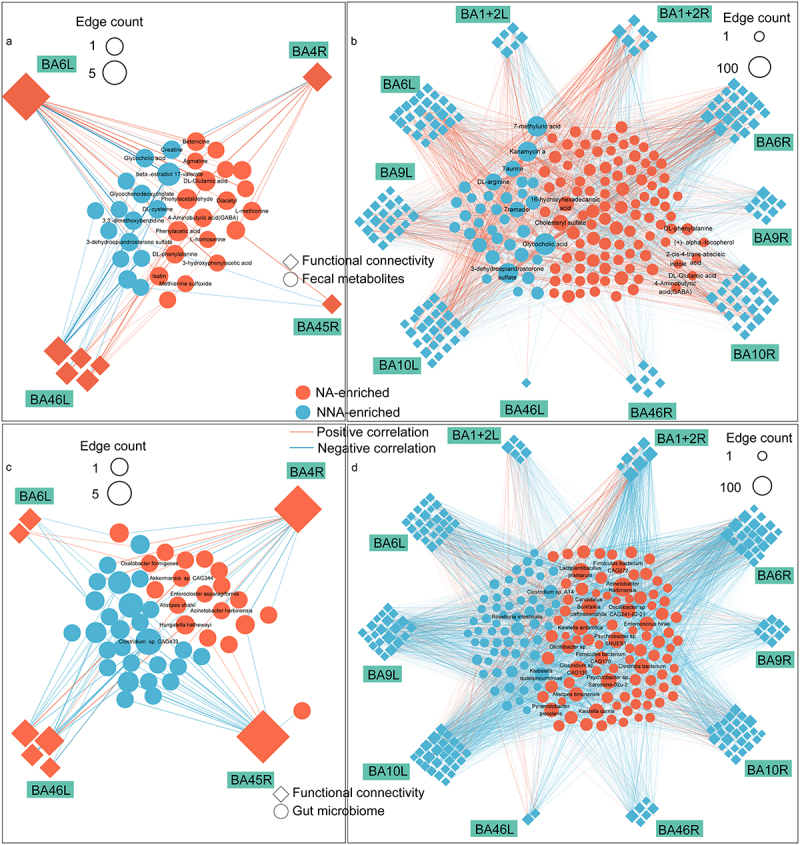
The correlation networks between fecal metabolomics/gut microbiota and brain functional connectivity.

Furthermore, correlation analysis revealed that most NA-related metabolites were significantly associated with NA-related brain functional connectivity. Most associations (2295/2466, 93.1%) appeared to promote the weakening of functional connectivity in the NAs and may be related to aging. However, some associations (171/2466, 6.9%) appeared to promote enhancement, which may contribute to longevity. Notably, some NA-enriched metabolites, such as GABA, glutamic acid, agmatine, and phenylalanine were positively correlated with NA-enhanced functional connectivity. However, some NA-depleted metabolites such as taurine and arginine were positively correlated with NA-weakened functional connectivity. Besides, we also found some NA-enriched metabolites were positively correlated with NA-weakened connectivity, such as GABA, indole, phenylalanine, α-tocopherol, and abscisic acid, suggesting a possible anti-aging effect on brain function (Figure 5a, b; Figure S7; Table S9).

(a) fecal metabolomics, and NA-enhanced functional connectivity. (b) fecal metabolomics, and NA-weakened functional connectivity. (c) gut microbiota, and NA-enhanced functional connectivity. (d) gut microbiota, and NA-weakened functional connectivity. The shade and opacity of connection lines corresponds to respective correlation coefficient.

Similarly, NA-related microbes were associated with differential NA-related functional connectivity. Likewise, for gut microbes, most (3512/3662, 95.9%) associations promoted weakened functional connectivity and some (150/3662, 4.1%) associations promoted their enhancement. Specifically, species related to fecal neuroactive compounds and antioxidants were associated with specific functional connectivity. For example, NA-enriched species, such as *Oxalobacter formigenes*, *Enterocloster asparagiformis*, *Hungatella hathewayi*, and *Eggerthella lenta* were positively correlated with NA-enhanced functional connectivity, such as BA46L-BA6L, BA46L-BA4R, and BA46L-BA6R. Some NA-depleted species, such as *Clostridium sp*. and *Roseburia intestinalis* were positively correlated with weakened connectivity in NAs, mainly in BA6R and BA9L (Figure 5c, d; Figure S8; Table S10).

### The potentially beneficial changes contributed to cognitive reservation in NAs

**Figure 6.**
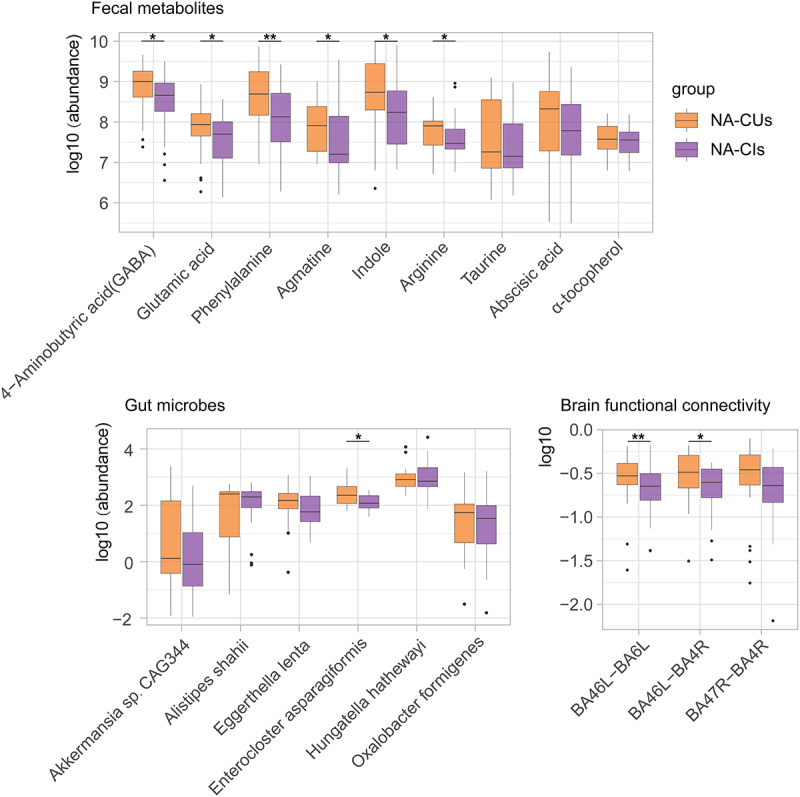
The potentially beneficial alterations between NA-CUs and NA-CIs.

We then divided 83 NAs into two subgroups, cognitively unimpaired (NA-CU, *n* = 33) group and cognitively impaired (NA-CI, *n* = 50) group, and compared the distribution of the abovementioned potentially beneficial modifications between the two subgroups. Our results showed that among the nine potentially beneficial metabolites, GABA, glutamic acid, phenylalanine, agmatine, indole, and arginine levels were significantly higher in NA-CUs than in NA-CIs. And among six potentially beneficial microbes, *Enterocloster asparagiformis* level was significantly higher in NA-CUs. In addition, among the potentially beneficial functional connectivity, BA46L-BA6L and BA46L-BA4R were significantly stronger in the NA-CUs group (Figure 6), indicating that potentially beneficial gut microbes may indirectly improve cognition by synthesizing neuroactive compounds and enhancing brain functional connectivity.

The boxplots show the distribution of potentially beneficial alterations between NA-CUs/NA-CIs. Differences in abundances between the two groups were tested by two-tailed Wilcoxon’s rank-sum test and “*” stands for *p*< .05, “**” stands for *p*< .01.

## Discussion

There is limited understanding of how gut microbes affect longevity through brain function. To our knowledge, this is the first study to integrate gut metagenome, metabolomics, and brain function to create a clear link between gut microbes and longevity. Interestingly, we found that interactions existed among the alternate gut microbiota, metabolites, and brain functional connectivity in nonagenarians, suggesting that the microbiota-gut-brain axis is not only involved in neurological diseases^19–21^ but also in longevity. Our results showed that several neuroactive substances modulated by *Enterocloster asparagiformis*, *Hungatella hathewayi*, *Oxalobacter formigene*, and anti-inflammatory substances were increased in nonagenarians, and were mainly associated with the enhancement of BA46L-BA6L, BA46L-BA4R, and BA47R-BA4R in brain functional connectivity. In addition, we found that most bacteria-derived neuroactive substances and enhanced functional connectivity were increased in the subgroup of nonagenarians with normal cognitive function, suggesting that changes in neuroactive metabolites and brain connectivity may contributed to cognitive reservation in the long-lived population.

Previous fecal metabolome studies have mainly shown increased levels of short-chain fatty acids (SCFA)^26^ and lithocholic acid derivatives in longevous individuals.^14^ Here, we report the upregulation of multiple neuroactive substances, such as GABA, agmatine, and indole, in a long-lived population. Several clinical trials and animal experiments have demonstrated the beneficial effects of neuroactive compounds.^18,27–32^ Studies have shown that GABA has various functions such as regulating synaptic transmission and promoting neuronal development.^27^ In addition, GABA prolongs the lifespan of C. elegans.^28^ Agmatine, a polyamine produced by the decarboxylation of arginine, is an endogenous neuromodulator that exerts antioxidant effects.^29^ Indole and isatin are bacteria metabolites from tryptophan.^18^ Although indoxyl sulfate has been reported to be increased in the blood of dementia patients,^33^ Indole derivatives have also been reported to have anti-inflammatory effects^30^ and can induce neurogenesis in adult mice by activating the AhR.^31^ These studies suggest that neuroactive substances may exert neuroprotective effects on longevity, including enhancing synaptic plasticity as well as antioxidant and anti-inflammatory activities. Interestingly, we also found that several neuroactive metabolites, such as taurine, were decreased in nonagenarians, which was consistent with previous study conducted by Cheng et al..^12^ Taurine is a neurotrophic factor with neuroprotective properties.^32^ We speculate that the reduction in taurine levels may reflect age-related health deterioration in long-lived people. Inflammation is a hallmark of aging that contributes to the pathogenesis of many age-related diseases.^34^ As a model of successful aging, people with longevity were found to contain high levels of anti-inflammatory molecules such as adiponectin and mitokines, representing a potent trigger of the anti-inflammatory response in longevity.^35,36^ In this study, we found several unique anti-inflammatory substances, such as α-tocopherol and abscisic acid, were increased in nonagenarians, contributing to the evidence that anti-inflammatory compounds promote longevity. Functional analysis of altered metabolites showed that the GABAergic synapse pathway in NAs was activated. Previous research shows that aging-induced damage to the GABAergic system makes the brain more susceptible to cognitive decline and damage caused by synaptic disease.^37^ Our finding suggest that the GABAergic synapse pathway remains active in long-lived individuals, providing insights into the neural mechanisms of longevity. In addition, functional analysis also showed that in pathway of arginine and proline metabolism, and taurine and hypotaurine metabolism, the neurotransmitter precursors arginine and cysteine were reduced in NAs, suggesting that arginine and cysteine can be supplemented to reduce the effects of aging on the nervous system.^38,39^

There have been several gut metagenomic studies on long-lived populations,^40–42^ in which increases in some gut microbes such as *Akkermansia*, *Alisipes*, and *Parabacteroides* have been reported. Consistent with previous research, our results showed *Akkermansia* and *Alisipes* increased in nonagenarians, suggesting the existence of common features in the gut microbes of long-lived individuals. *Akkermansia* is a potential probiotic that has been widely studied in recent years,^43^ and *Alistipes* has been reported to protect against certain diseases, including liver fibrosis and cardiovascular disease.^44^ In addition, we found, for the first time, that *Enterocloster asparagiformis*, *Hungatella hathewayi*, and *Oxalobacter formigenes* were increased in long-lived individuals. *Enterocloster asparagiformis* has been reported to be an SCFA-producing species^45^ and is negatively correlated with waist circumference.^46^ *Hungatella hathewayi* protects mice against intracranial aneurysm formation and rupture.^47^ *Oxalobacter formigenes* has been reported to be capable of degrading oxalate in the gut lumen and protecting against kidney stones.^48^ We also found that a unique potentially harmful bacteria *Sutterella* was depleted in nonagenarian, which was reported to be associated with ulcerative colitis and intestinal biopsy.^49,50^ Extensive metabolic interactions have been reported between gut microbes and their host.^51^ Functional analysis of microbes revealed that the enrichment of metabolites in NAs was associated with gut microbiota-mediated multiple function, reveal complex and diverse relationships between microbiota and metabolites in NAs. Microbial characteristics in long-lived populations have been previously associated with certain beneficial metabolites.^1,12,13^ For example, *Odoribacteraceae* isolated from the feces of a long-lived Japanese population can synthesize isoallolithocholic acid, which shows antibiotic activity against pathogenic bacteria.^14^ In this study, some gut microbes enriched in long-lived populations, such as *Enterocloster asparagiformis*, *Hungatella hathewayi*, and *Oxalobacter formigenes*, synthesized neuroactive substances, including GABA, glutamic acid, indole, agmatine, and phenylalanine. The strength of the synthetic ability positively correlated with the level of the corresponding neuroactive substances. Our findings demonstrated the beneficial effects of the longevity-related species that we focused on in our research and further suggested that healthy aging could be promoted by supplementing longevity related potential beneficial species or neuroactive compounds.

We examined brain function in nonagenarians using fNIRS for the first time, which is suitable for functional neuroimaging studies of long-lived individuals because it is simple, fast, and noiseless. To date, only one study has assessed the brain function of 46 near-centenarians using functional magnetic resonance imaging and reported that the connectivity of the FPCN was stronger in near-centenarians.^52^ Our results showing that the connectivity between BA46L-BA6L was enhanced in nonagenarians was consistent with their results, since both BA46 (dorsolateral prefrontal cortex, DLPFC) and BA6 (premotor cortex, PMC) are parts of FPCN. We also found that the connectivity between BA46L-BA4R and BA47R-BA4R was stronger in nonagenarians. The connection between BA46 (dorsolateral prefrontal cortex, DLPFC) and BA4 (primary motor area, M1) is called “DLPFC – M1 interaction” and is important for fine movement of the hands.^53^ The decline of interaction between DLPFC and M1, especially interhemispheric, accounted for bimanual performance deficits in older people.^54^ The connection between BA47 (inferior frontal gyrus, IFG) and BA4 (primary motor area, M1) is involved in motor control.^55^ We further explored the relationship between gut microbes, metabolites and brain functional connectivity, and found that some increased neuroactive compounds and species that can synthesize neuroactive compounds in nonagenarians were positively correlated with multiple brain functional connectivity, suggesting that they may play a role in promoting the strengthening and delaying the weakening of some connectivity. Some of the aforementioned metabolites and microbes worked together on the same functional connectivity, such as *Enterocloster asparagiformis* and GABA, which functioned together to enhance BA46L-BA6L connectivity, and *Hungatella hathewayi*, glutamic acid, and agmatine, which worked together to enhance BA46L-BA4R connectivity, suggesting that gut microbes in long-lived individuals may affect the strength of brain functional connectivity by modulating neuroactive substances.

Because connectivity in the FPCN is associated with better cognitive performance in near-centenarians, we further explored the relationship between microbes and cognition in nonagenarians. Our results showed that distribution of potentially beneficial species *Enterocloster asparagiformis* differed significantly between normal-aging nonagenarians and cognitively impaired nonagenarians, and most neuroactive substances and enhanced connectivity were increased in the normal-aging subgroup, suggesting that microbial characteristics in the long-lived population may improve cognition by increasing connectivity strength and neuroactive metabolites.

However, these results should be interpreted with caution, as they are purely associative and represent a first step in human research. To further elucidate the actual interactions and mechanisms of these key species along the metabolism pathway on improving brain function in longevity, future studies will more deeply explore through molecular biology and animal experiments.

In conclusion, our study identified potentially beneficial mediators of the microbiota-gut-brain axis in longevity. We found that unique gut microbes in nonagenarians could enhance brain functional connectivity by producing neuroactive compounds and further improving cognitive function. Our findings provide potential intervention targets for extending the human lifespan and can help promote healthy aging.

## Participants and methods

### Participants recruitment

We recruited 187 participants from Changsha, Hunan province, China in March 2023. Individuals who had digestive system diseases or had taken antibiotics or probiotics within the previous two weeks were excluded. Finally, 164 participants, including 83 NAs and 81 NNAs, were eligible for this study. Individuals live independently, and did not take special diets or medications. The MMSE and CDR were used to assess the cognitive function of the participants. The trained investigators in this study included interviewers and neurologists who had received consistent training in neuropsychological assessment. First, an interviewer used the MMSE to assess global cognitive function, with the cutoff values for CI set according to the criteria reported in a previous Chinese population study (illiterate participants, ≤17 points; primary-education participants, ≤20 points; junior secondary school or higher education participants, ≤24 points).^56^ Another interviewer conducted the CDR assessment, with the necessary information obtained through a semi-structured interview with the participant and a reliable informant. A CDR ≥ 0.5 was used to designate CI.^57^ Individuals who did not meet the CI criteria were classified as CU. The ethics committee of Xiangya Hospital, Central South University approved this study, and all participants or their guardians provided written informed consent before sample collection (No.202005124).

### Fecal sample collection

Approximately 2 g of fecal sample was collected from each participant using a Longseegen Stool Storage Kit (Cat. No. : LS-R-P-007, Longsee Biomedical Corporation, Guangzhou, China), according to the manufacturer’s instructions, and then stored in a refrigerator at −80°C.

### Metabolome profiling of fecal samples

#### Metabolite extractions

One milliliter of the cold extraction solvent methanol/acetonitrile/water (2:2:1, *v*/*v*) was added to 80 mg of the sample and vortexed. The samples were then incubated on ice for 20 mins, then centrifuged at 14,000 g for 20 mins at 4°C. The supernatant was then collected and flowed through a 96-well protein precipitation plate; the elution was collected and dried in a vacuum centrifuge at 4°C. The samples were then re-dissolved in 100 μL acetonitrile/water (1:1, *v*/*v*) solvent and transferred to LC vials for LC-MS analysis.

#### LC-MS/MS analysis

The extracts were analyzed using a quadrupole time-of-flight mass spectrometer (Sciex TripleTOF 6600, USA) coupled with hydrophilic interaction chromatography via electrospray ionization (HILIC, ACQUIY UPLC BEH Amide column, 2.1 mm × 100 mm, 1.7 µm). Column temperature was set to 25°C for separation of HILIC and 2 µL of gradient was injected at a flow rate of 0.4 mL/minute. The mass spectrometer was operated in both negative and positive ionization modes. The ESI source conditions were set as follows: Ion Source Gas1 (Gas1) as 60, Ion Source Gas2 (Gas2) as 60, curtain gas (CUR) as 30, source temperature: 600°C, IonSpray Voltage Floating (ISVF) ± 5500 V.

XCMS software were used for raw peaks exacting, including the data baselines filtering and calibration of the baseline, peak alignment, deconvolution analysis, peak identification and integration of the peak area. The missing values of raw data were filled up by KNN (Kth nearest neighbor) method.

### Metagenomic sequencing and data analysis

#### Metagenomic sequencing

Total DNA was extracted using a Magnetic Soil and Stool DNA Kit (TIANGEN, China) according to the manufacturer’s instructions. Qualified examples were used to create the libraries. Sequencing libraries were generated utilizing the NEBNext® UltraTM DNA Library Prep Kit for Illumina (NEB, USA) in accordance with the manufacturer’s instructions, then index codes were added to assign sequences to specific samples. Using the cBot Cluster Generation System, index-coded samples were clustered in accordance with the manufacturer’s recommendations. Library preparations were sequenced on an MGISEQ-T7 (MGI, China) after cluster creation, and 150 bp paired-end reads were produced.

#### Data analysis

The data generated from the BGI platform were used for bioinformatics analysis. Raw data obtained from the BGI sequencing platform were preprocessed using fastp to acquire clean data for subsequent analyses using default parameters. Clean data were assembled using MEGAHIT software. Contigs were predicted using the Prodigal software and translated into amino acid sequences. For the ORF prediction results, CD-HIT software was used to generate a non-redundant gene catalog with default parameters: 95% identity and 90% coverage were used for clustering, and the longest sequence was selected as the representative sequence. Using the bowtie2 software, the clean reads of each sample were aligned to the non-redundant gene catalog with a cutoff identity 95%. DIAMOND software was used to blast the gene catalog to NR (Version:2021.11) and KEGG databases to obtain species and function information. The best BLAST hit of each sequence was used for subsequent analyses.

#### α-diversity and β-diversity

The α-diversity was estimated according to species count and the Shannon index. Species counts were calculated by tallying non-zero species in each sample, and the Shannon index was calculated based on the species abundance profile of each sample The β-diversity was calculated based on Bray – Curtis dissimilarity using the R vegan package.

### fNIRS data acquisition and processing

#### Acquisition

The participants underwent a 5-min session using a multichannel fNIRS system (NirSmartII-3000A, Hui Chuang, China) with two wavelengths (730 nm, 850 nm) at a sampling rate of 11 Hz. The measuring channels consisted of 14 sources and 14 detectors, which were designed to form 35 channels covering the frontal, temporal, and parietal lobes. All participants were instructed to remain still and awake with their eyes open during the entire session. The 3D positions (MNI standard coordinates) of the channels were calculated using a 3D digitizer (PATRIOT, Polhemus), which was based on a four-point positioning algorithm in 3D space, relying on the received signal strength indication. The fNIRS channels were then mapped to 13 ROI according to their MNI coordinates.

#### Data preprocessing

NirSpark (Huichuang, China) was used to preprocess the obtained signals. Firstly, artifacts were detected using a time window of 0.5 seconds and two thresholds. The first threshold was the standard deviation of the entire data multiplied by a coefficient and was set to 6. The second threshold had a fixed value of 0.5. If the standard deviation of the time window exceeded the first threshold or the peak amplitude exceeded the second threshold, the data of this time window were defined as motion artifacts and corrected using spline interpolation. Secondly, band-pass filtering of the data between 0.01–0.2 Hz was performed to remove instrumental and physiological noise. Third, the artifact-free and filtered data were converted into HBO concentration changes according to the modified Beer-Lambert law. Finally, the HBO in 10–310 s after the onset of detection were extracted for further analysis.

#### Functional connectivity network analysis

The Pearson’s correlation coefficient was calculated to determine the functional connectivity between each pair of measurement channels. Subsequently, Fisher’s r-to-z transformation was applied to convert the correlation coefficients to z-scores to improve the normality. In the ROI-based analysis, the functional connectivity of the 13 ROIs’ internal channels was averaged to obtain ROI-based z-scores and generate a 13 × 13 ROI-to-ROI connectivity (RRC) matrix for each participant.

### Statistical analysis

#### Multivariate analysis

Multivariate statistical analyses were used to discriminate between NAs and NNAs. PCA of subject-level RRC matrixes was performed using the R *ade4* package. The dbRDA was performed based on Bray – Curtis dissimilarity in the fecal metabolome, gut microbial composition, and functional profile using the R *vegan* package.

#### Procrustes analysis

Procrustes analysis was performed using the R *vegan* package, and the Procrustes *p*-value was generated based on 999 permutations.

#### PERMANOVA tests

PERMANOVA was conducted on the Bray – Curtis dissimilarity of the species abundance and fecal metabolite profiles of the samples as well as subject-level RRC matrixes to assess the effect of age characteristics using 999 permutations in the R *vegan* package.

#### Cluster analysis

K-means cluster analysis was conducted using the R *stats* package.

#### Association analysis

Spearman’s correlation was used to investigate the association between gut microbes and fecal metabolites, the association between species-level gene abundance and corresponding neuroactive compounds, and the possible effects of each NA-related fecal metabolite and gut species on the significant functional connectivity in long-lived brains. Gut species and fecal metabolites with*p* < .05 were associated with alterations in the functional connectivity of the NAs.

#### Hypothesis test and multiple test correction

The differential abundance of gut microbial composition was tested by LEfSe analyses, and gut microbial function, fecal metabolites, and species-level gene abundance were tested using a two-tailed Wilcoxon’s rank-sum test between NAs/NNAs and NA-CUs/NA-CIs. The functional connectivity strength of fNIRS between NAs/NNAs was tested using a general linear model (GLM), with education years as covariates. The functional connectivity strength of fNIRS was tested using two-sample *t*-tests between NA-CUs/NA-CIs. The false discovery rate (FDR) was adjusted using the Benjamin-Hochberg method.

## Supplementary Material

Supplementary Table.xlsx

Supplementary Figure.docx

## Data Availability

Data will be available on request.
