# Supplementary material for: Integrated analysis of gut metabolome, microbiome, and brain function reveal the role of gut-brain axis in longevity
Source: Gut Microbes. 2024 Mar 28;16(1):2331434. doi: 10.1080/19490976.2024.2331434 (PMC10984123; doi:10.1080/19490976.2024.2331434)
Supplement: Supplementary Figure.docx [file KGMI_A_2331434_SM8780.docx]

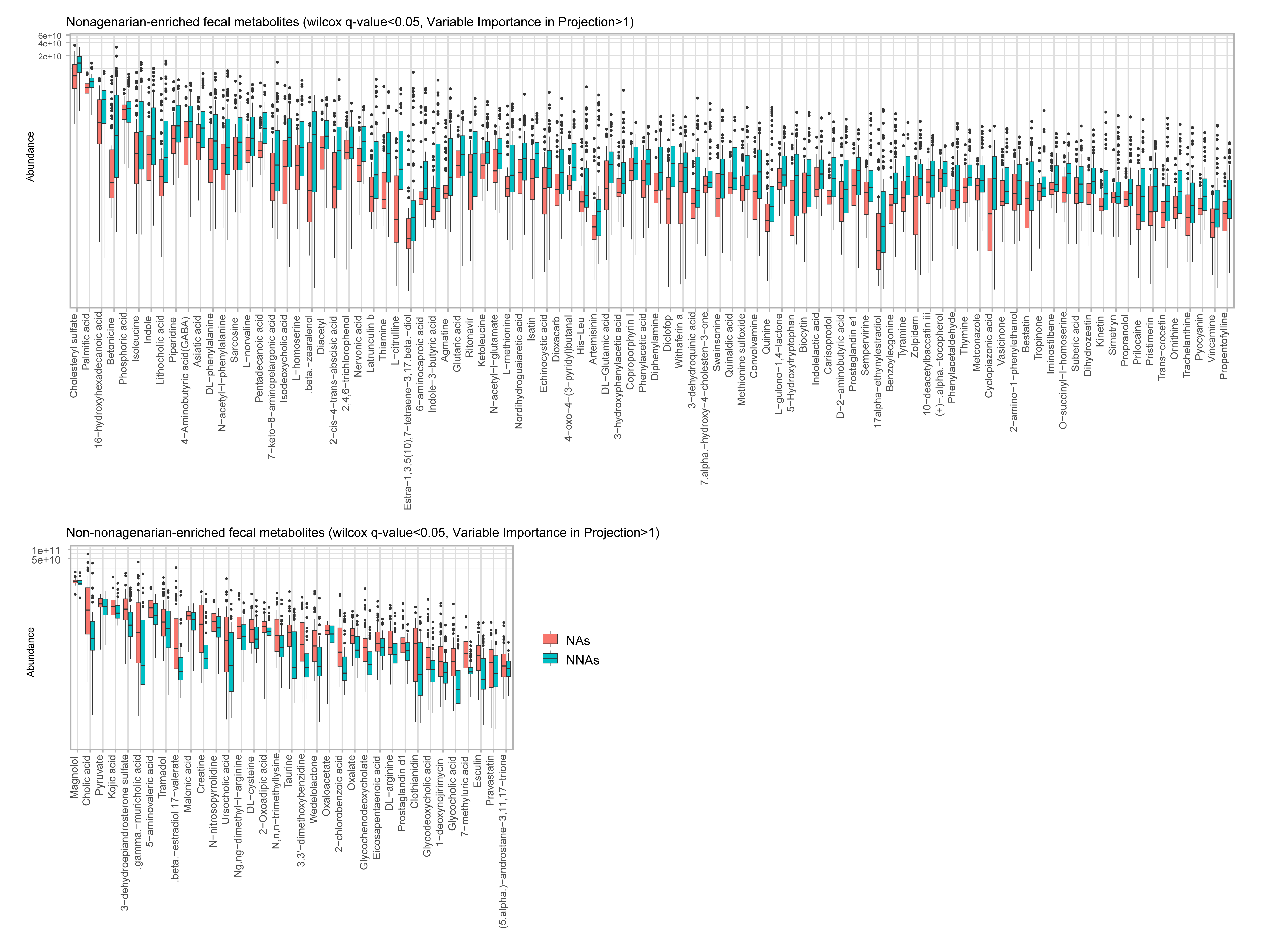


**Supplementary Figure 1. Differences in abundances of fecal metabolites between** **nonagenarians and non-nonagenarians**


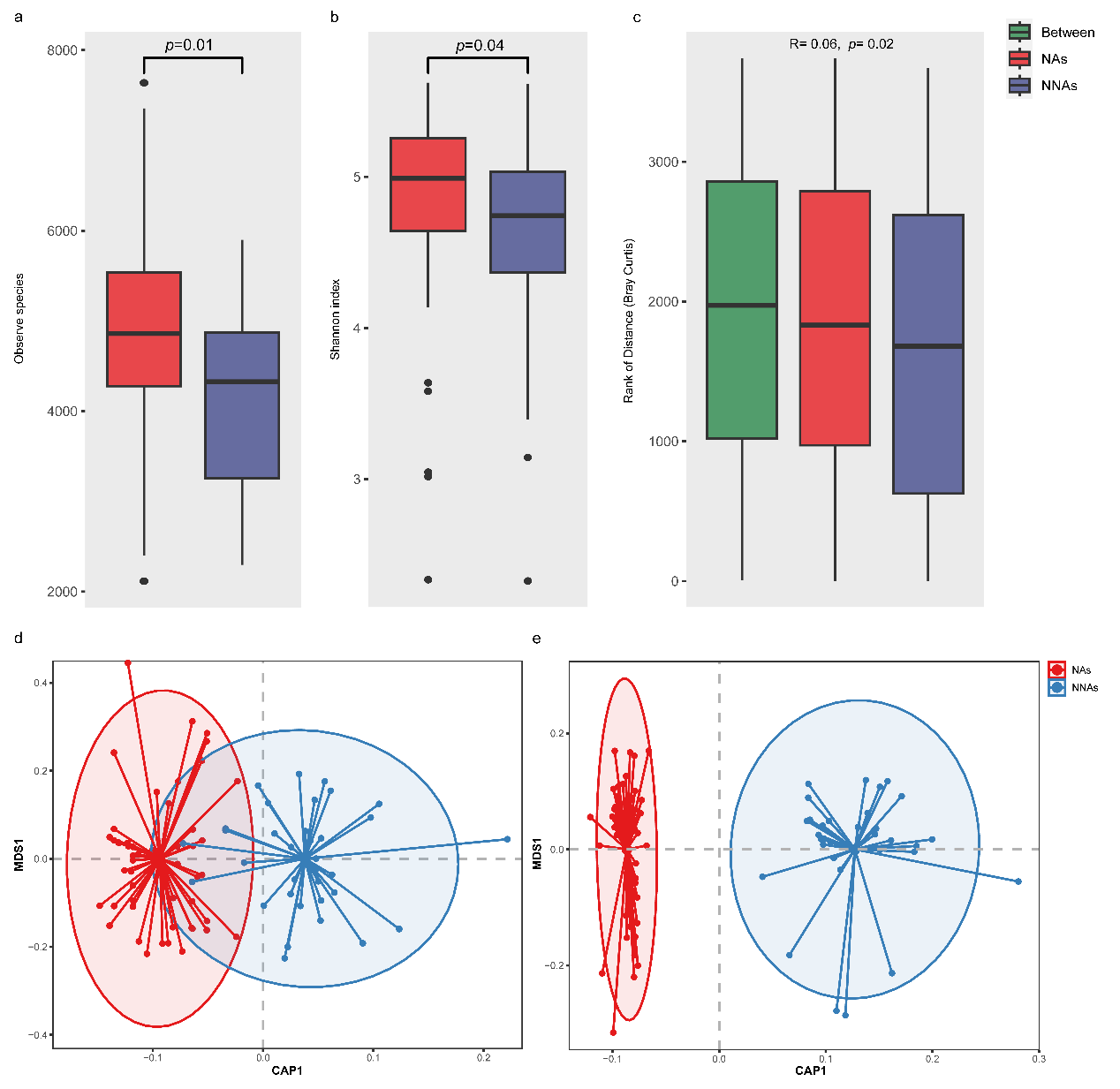


**Supplementary Figure 2. Characteristics in microbial composition and function in nonagenarians.** (a) Boxplots showing the Shannon index in NAs and NNAs; (b) Boxplots showing the intra-group beta diversity in NAs and NNAs. dbRDA revealed a significant difference in the gut microbial species composition; (c) functional profile (d) between NAs and NNAs.


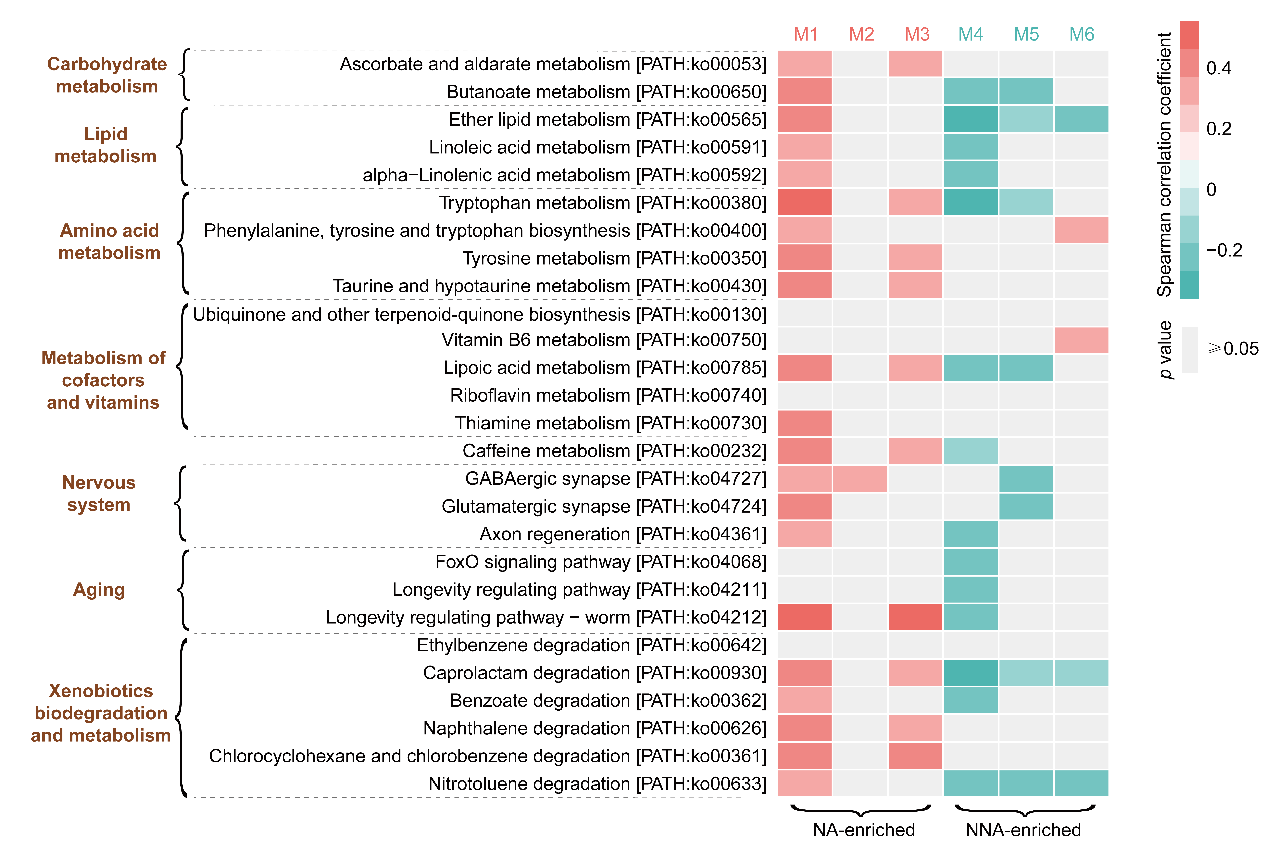


**Supplementary Figure 3. Relationships between specific gut microbial functions and the concentration of NAs-related fecal metabolites.** The heatmap displays the significant Spearman correlation coefficients between functional pathways and fecal metabolite clusters. Details of metabolite clusters were shown in Supplementary Table 2.


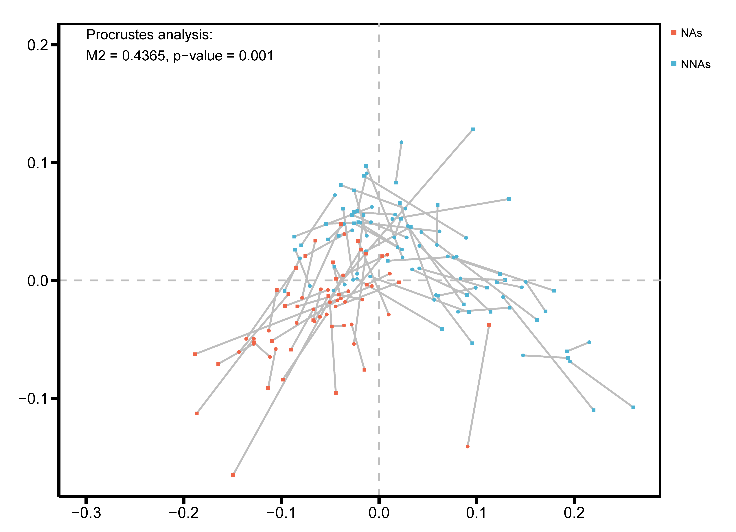


**Supplementary Figure 4. Relationships between gut microbiota and fecal metabolome.** Subject-level microbiota and metabolome data are shown as circles and squares, respectively, and connection lines reflect the microbiota and metabolome data for one individual.


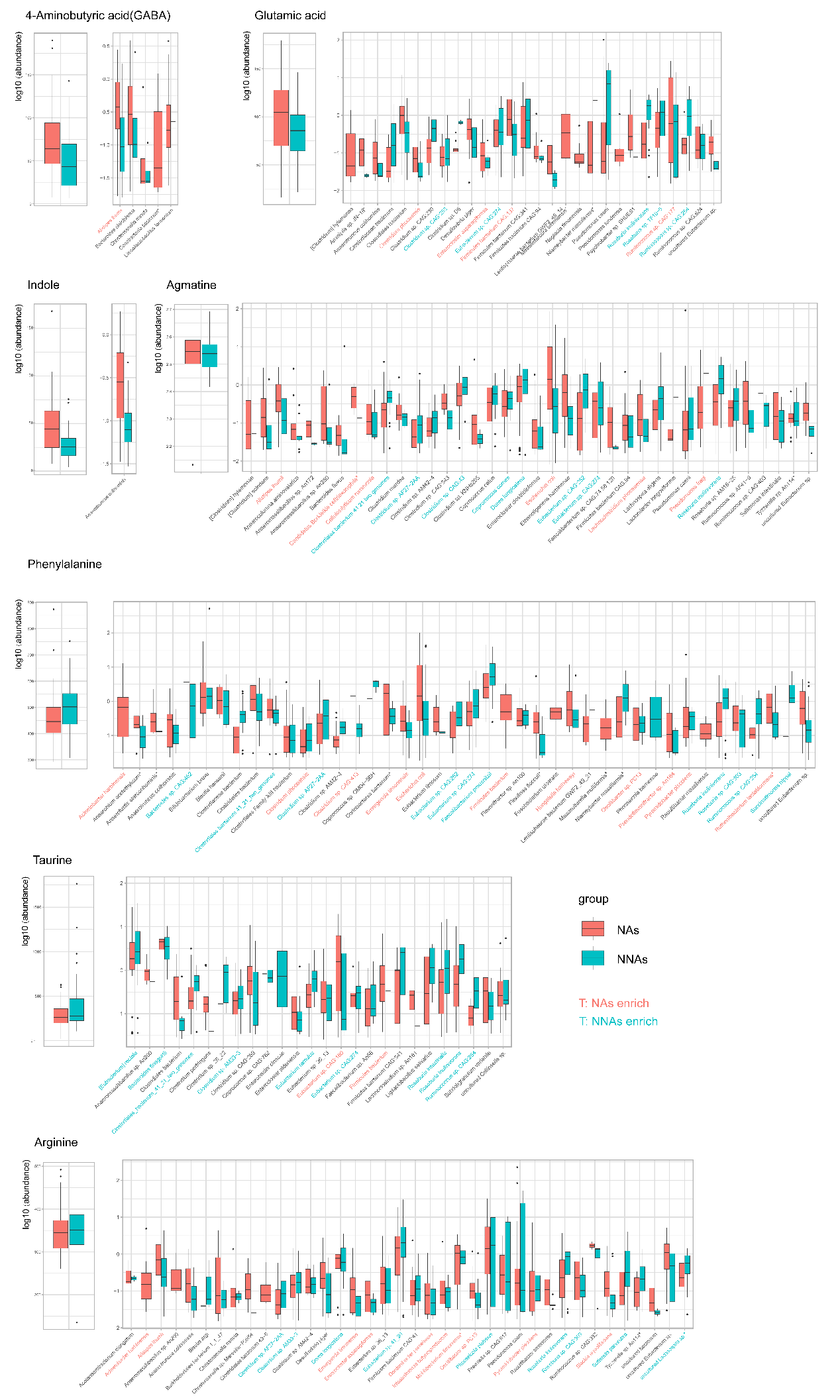


**Supplementary Figure 5. Distribution of the key synthetases involved in the biosynthesis of neuroactive metabolites.** Abundance of key synthetase-encoding genes in NAs and NNAs and in corresponding species


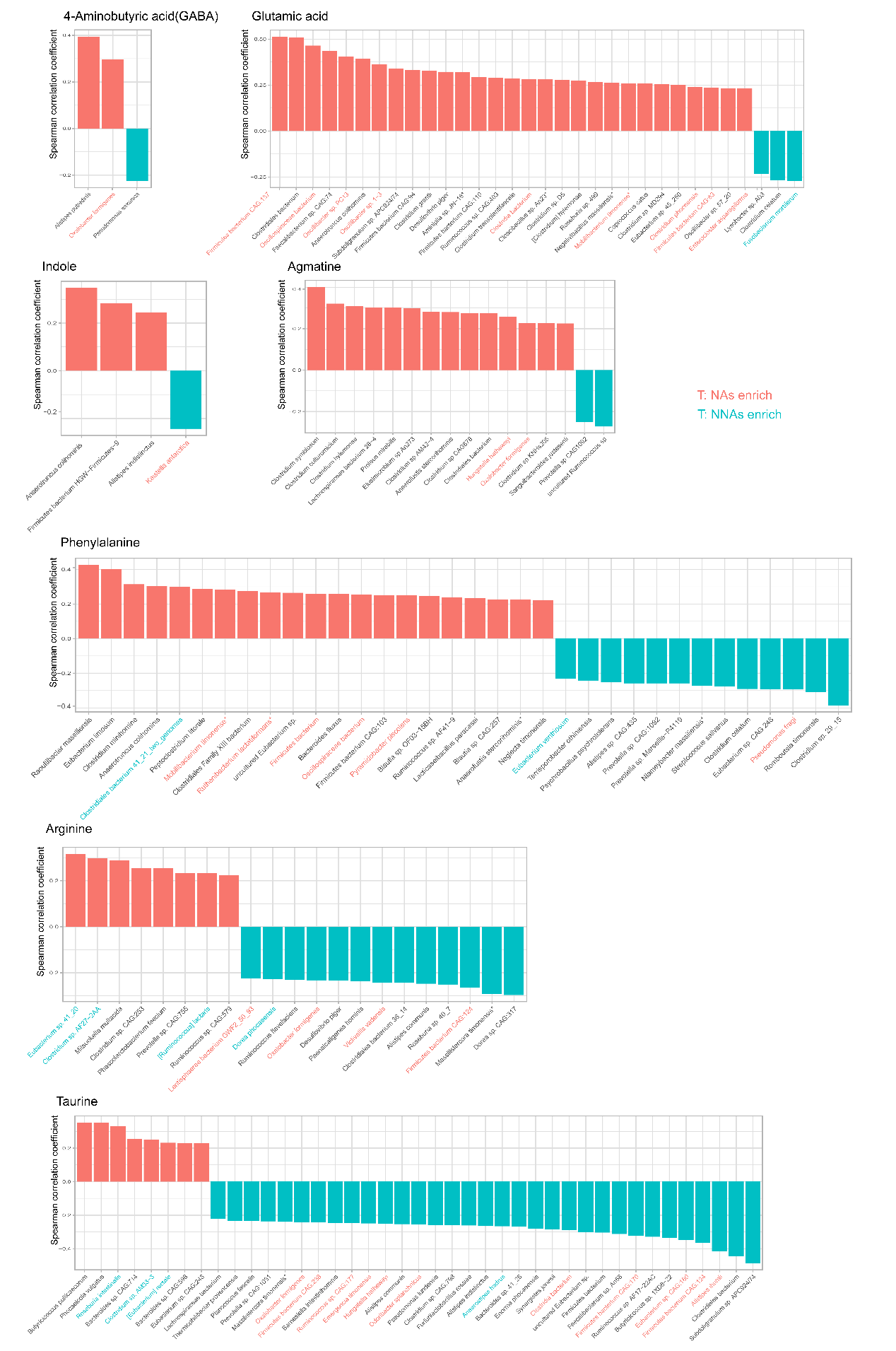


**Supplementary Figure 6. The relative abundances of species-level abundance of key synthetases encoding genes and their correlations with corresponding fecal metabolites**


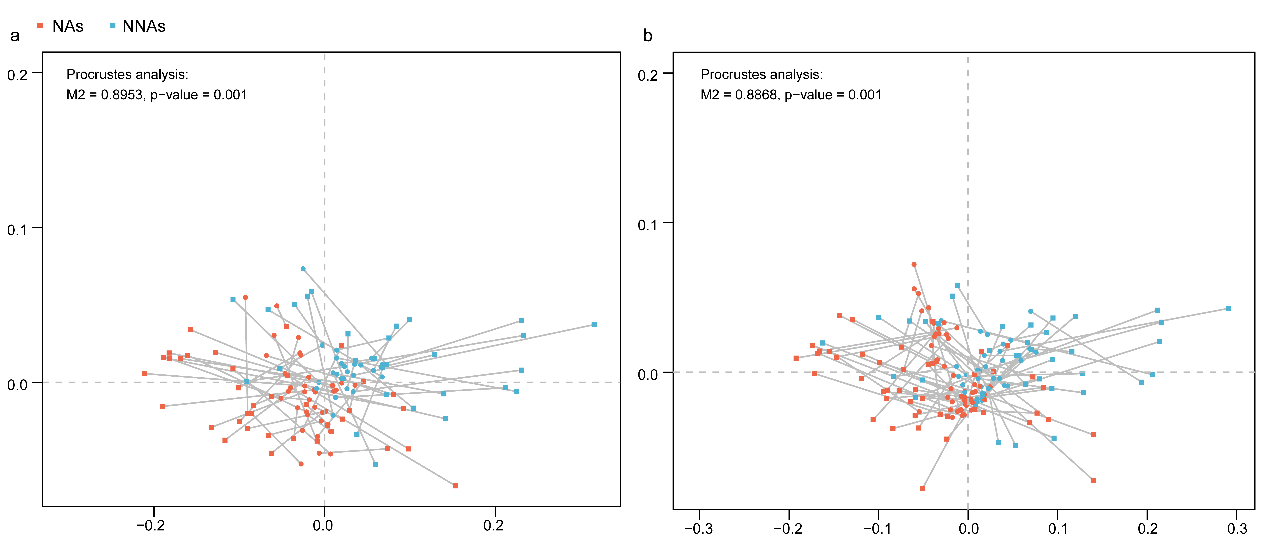


**Supplementary Figure 7. Relationships between gut microbiota/fecal metabolome and brain functional connectivity.** Subject-level microbiota/metabolome data and brain functional connectivity are shown as circles and squares, respectively, and the connection lines reflect the microbiota/metabolome data and functional connectivity in one individual.


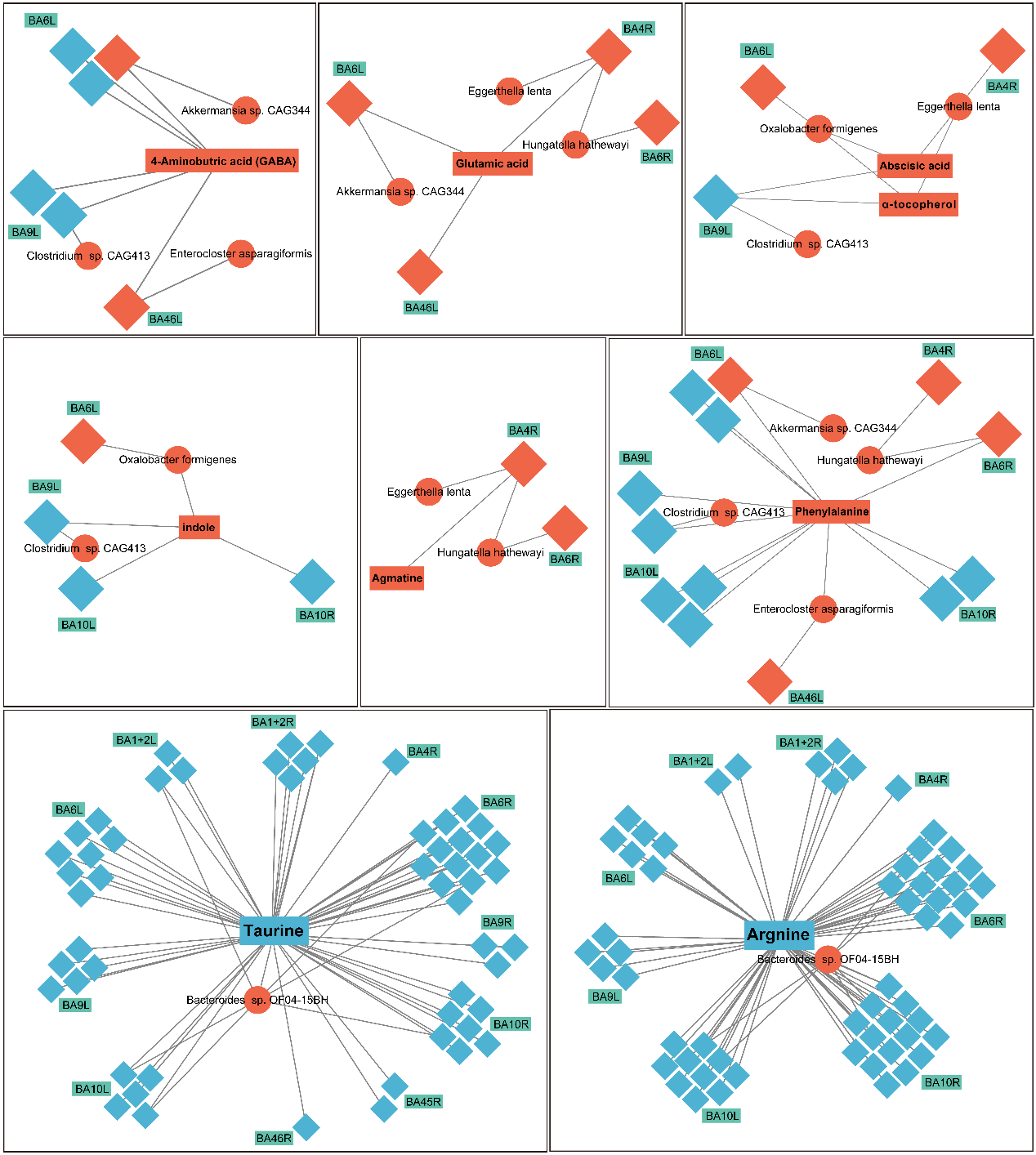


**Supplementary Figure 8. The correlation networks between potentially beneficial metabolites/microbes and brain functional connectivity.** The connection lines represent significant Spearman correlations (*p*< 0.05) between potentially beneficial metabolites, gut microbes, and functional connectivity.
